# Comparative Efficacy and Safety of the β3-Adrenoceptor Agonist Vibegron for Urgency and Mixed Urinary Incontinence: A Post Hoc Analysis of a Randomized, Double-Blind, Placebo-Controlled Phase 3 Study

**DOI:** 10.7759/cureus.71106

**Published:** 2024-10-08

**Authors:** Masaki Yoshida, Makoto Ikeda, Shigeki Nawata, Shinji Nagai, Shinichi Kubono, Takashi Uno, Shotaro Maeda

**Affiliations:** 1 Department of Urology, Sakurajyuji Hospital, Kumamoto, JPN; 2 Medical Affairs, Kyorin Pharmaceutical Co. Ltd., Tokyo, JPN; 3 Data Science Group, Clinical Data Science &amp; Affairs, Kyorin Pharmaceutical Co. Ltd., Tokyo, JPN; 4 Medical Research Department, Kissei Pharmaceutical Co. Ltd., Tokyo, JPN

**Keywords:** mixed urinary incontinence, overactive bladder, post hoc analysis, randomized controlled trial, urgency urinary incontinence, vibegron, β3-adrenoceptor agonist

## Abstract

Objective

To compare the efficacy of vibegron for urgency urinary incontinence (UUI) and mixed urinary incontinence (MUI) in patients with overactive bladder (OAB).

Methods

We performed a post hoc analysis of a phase 3 study of vibegron in Japanese patients with OAB. Based on the patterns recorded in the three-day bladder diary, only female patients were categorized into UUI and MUI populations. The primary endpoint was the change in the mean micturition number per 24 hours from baseline to week 12. Other endpoints included the mean changes in urgency episodes per 24 hours, incontinence episodes per 24 hours, voided volume per micturition, and the number of nocturia episodes per night. The proportion of urinary incontinence (UI) normalization, quality of life (QOL) as measured by the King’s Health Questionnaire (KHQ), and patient satisfaction level as assessed by the Patient Global Impression (PGI) were investigated.

Result

Data from a UUI population (vibegron 50 mg, n = 237; vibegron 100 mg, n = 231; placebo, n = 237) and an MUI population (vibegron 50 mg, n = 70; vibegron 100 mg, n = 77; placebo, n = 78) were analyzed. Vibegron 50 mg and 100 mg significantly improved the change in micturition number per 24 hours from baseline to week 12. Compared to those of patients receiving a placebo, all other endpoints of urinary parameters were significantly improved in patients receiving vibegron, except for nocturia in the vibegron 50 mg group in both populations. In the vibegron 50 mg, vibegron 100 mg, and placebo groups, the proportions of those who attained normalization of UI were stratified by the UUI (51.5%, 57.6%, and 44.7%, respectively) and MUI (40.0%, 41.6%, and 20.5%, respectively) populations. Furthermore, both vibegron doses improved KHQ scores in both populations, and PGI improvement was significantly higher than that in the placebo group in both populations.

Conclusion

This post hoc analysis demonstrated that vibegron has equivalent efficacy and safety in the MUI and UUI populations. The results of this study provide important information for the treatment of MUI.

## Introduction

Urinary incontinence (UI) is characterized by involuntary urine leakage; its prevalence increases with age, with 9-39% of women older than 60 years experiencing UI episodes at least once a day [1]. UI has a significant impact on patients’ quality of life (QOL) [2]. In Japan, the latest epidemiological survey showed that UI has a negative impact on daily life and occurs predominantly in women [3]. Several organizations, including the International Continence Society, have classified UI into several types, including urgency urinary incontinence (UUI), stress urinary incontinence (SUI), mixed urinary incontinence (MUI), functional incontinence, and overflow incontinence [4,5]. MUI is characterized by the symptoms of both UUI and SUI and manifests as involuntary urine leakage that is associated not only with urgency, but also with physical exertion, effort, sneezing, and coughing. UUI is a symptom of an overactive bladder (OAB). In cases of MUI wherein UUI is the predominant subtype, pharmacotherapeutic agents for OAB have been used as therapeutic options [6].

β_3_-adrenoceptor agonists are a pharmacotherapeutic option for patients with OAB. In Japan, two β_3_-adrenoceptor agonists, mirabegron and vibegron, have been approved and are commercially available. In clinical trials, vibegron significantly improved symptoms compared to placebo and was generally well tolerated [7]. Furthermore, non-clinical studies have shown that vibegron has high β_3_-adrenoceptor selectivity [8,9]. Although vibegron improves UUI symptoms regardless of the severity [10], the efficacy and safety of vibegron for MUI remain unelucidated.

Therefore, using a post hoc analysis of data from a phase 3 study of vibegron, we evaluated the efficacy and safety of vibegron in patient populations subclassified into UUI and MUI based on information from the bladder diary.

## Materials and methods

Study design and participants

The vibegron phase 3 study (JapicCTI-152936) was a placebo-controlled, multicenter, randomized, double-blind study, and the results have been described [7]. The study was conducted from July 2015 to June 2016. It was carried out in accordance with the Declaration of Helsinki, International Council for Harmonization Good Clinical Practice Guidelines, and was approved by the Institutional Review Board at each study site. Prior to the commencement of any study-related procedures, all participants provided their written informed consent. The inclusion criteria were as follows: an average daily urinary frequency ≥8.0, an average daily UUI episode frequency ≥1, or an average daily urinary urgency episode frequency ≥1. Additionally, the total number of UUI episodes should exceed half the total number of incontinence episodes. These criteria ensured that the study sample comprised patients with urgency-predominant UI. The exclusion criteria included residual urinary volume >100 mL, interstitial cystitis, bladder cancer, urinary tract infection, and inability to independently access a toilet for micturition. The participants were then randomly assigned to four groups: vibegron (50 mg or 100 mg once daily), placebo, or imidafenacin (0.1 mg twice daily), and each group was followed up for 12 weeks. From this patient population, female patients with UI episodes who were taking either vibegron (50 mg or 100 mg once daily) or a placebo were enrolled in this post hoc analysis.

Classification of UUI and MUI population

In the phase 3 study of vibegron, details recorded in the bladder diary included episodes of voiding, urgency, incontinence, and voided volume, along with their respective times, which were recorded for three consecutive days. Patients with at least one episode of incontinence during the three-day period were included in the analysis. Accordingly, based on the information from the bladder diary, we classified patients who experienced incontinence episodes only with urgency as the UUI population. Patients having incontinence both with and without urgency were classified as the MUI population. As patients with overflow incontinence and those with functional incontinence were excluded from the study, incontinence in the MUI population was considered to include both UUI and SUI.

Evaluation of efficacy and safety

In both the UUI and MUI populations, changes in these urinary parameters from baseline (week 0) to week 12 of treatment were calculated and compared between the vibegron (50 mg and 100 mg) and placebo groups. The primary endpoint was the mean change in micturition number per 24 hours from baseline to week 12. The other endpoints included mean changes in the number of urgency episodes per 24 hours, number of UI episodes per 24 hours, voided volume per micturition, and nocturia episodes per night. The proportion of UI normalization was calculated as the percentage of patients without incontinence episodes at week 12 in each group.

The QOL related to UI was assessed using the King’s Health Questionnaire (KHQ) [11] to evaluate changes from baseline to week 12. Additionally, patient satisfaction was measured at week 12 using the Patient Global Impression (PGI) scale [12], which was graded on a seven-point scale: 1, very much improved; 2, much improved; 3, minimally improved; 4, no change; 5, minimally worse; 6, much worse; and 7, very much worse. PGI scores of 1 and 2 were defined as “very much satisfied,” whereas PGI scores of 1, 2, and 3 were defined as “satisfied.”

Safety analyses were performed for each population in the full analysis set (FAS) and evaluated based on the most common treatment-emergent adverse events (TEAEs) for which a causal relationship to the study drug could not be ruled out.

Statistical analysis

Changes in the efficacy endpoints from baseline to week 12 were analyzed for the vibegron (50 mg and 100 mg) and placebo groups in the UUI and MUI subpopulations. This analysis used a constrained longitudinal data analysis model that considered adjustment factors (prior OAB treatment history and urgency incontinence status), except for baseline mean micturition. The least-squares mean (LS mean) and two-sided 95% confidence intervals (CIs) were calculated using this method [13]. To evaluate the improvement with vibegron over that with placebo, the differences in the efficacy endpoints between the vibegron and placebo groups were compared using the same constrained longitudinal data analysis model. The Chi-squared test was used to compare the proportion of UI normalization and the degree of symptom improvement based on the PGI scores between the vibegron and placebo groups. All analyses were conducted using SAS software (version 9.4; SAS Institute, Cary, NC, USA); the analyses were performed using two-sided tests with a significance level of 0.05. Adverse events were classified using MedDRA/J version 17.1 (Medical Dictionary for Regulatory Activities, Japanese Edition).

## Results

Patient characteristics

**Figure 1:**
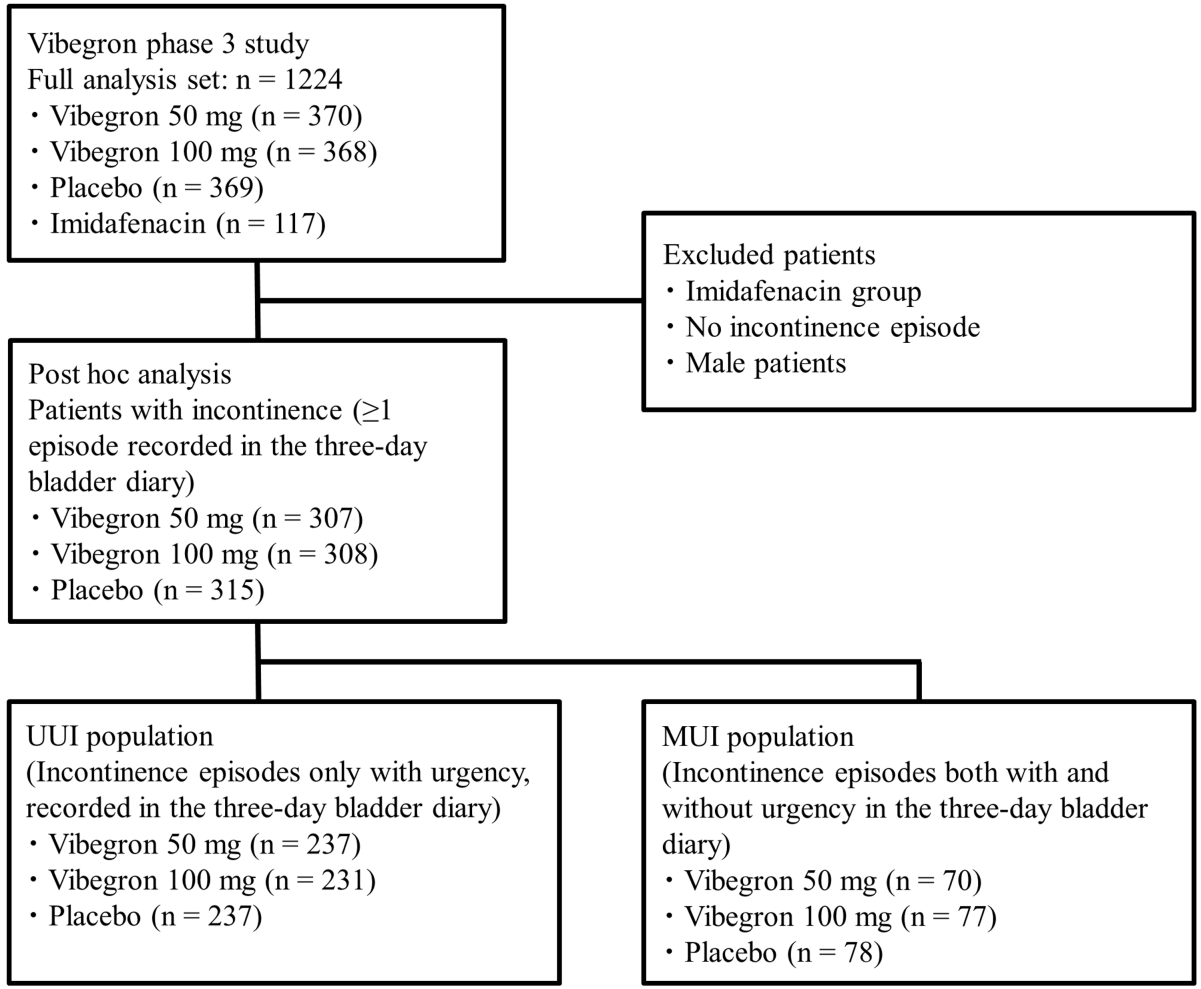
Study population of defined UUI and MUI populations. UUI: Urgency urinary incontinence; MUI: Mixed urinary incontinence

Of the 1,224 patients in the FAS population in the original trial, after excluding the imidafenacin group (reference group), patients without UI episodes, and male patients, the present analysis included 930 patients with UI episodes who were assigned to the vibegron (50 mg and 100 mg) and placebo groups (Figure 1). The vibegron 50 mg, vibegron 100 mg, and placebo groups comprised 237, 231, and 237 patients, respectively, in the UUI population, and 70, 77, and 78 patients, respectively, in the MUI population. The baseline clinical characteristics of the three participant groups in each population were similar, and no statistically significant intergroup difference was observed, except in the KHQ domain 5 in the UUI population, as well as the mean micturition episodes per 24 hours and KHQ domain 4 in the MUI population (Table 1).

**Table 1:** Demographics and baseline clinical characteristics of the participants. Data are mean (SD) BMI: Body mass index; OAB: Overactive bladder; KHQ: King’s Health Questionnaire; UUI: Urgency urinary incontinence; MUI: Mixed urinary incontinence

	UUI population							MUI population						
	Vibegron 50 mg		Vibegron 100 mg		Placebo		p-value	Vibegron 50 mg		Vibegron 100 mg		Placebo		p-value
	(n = 237)		(n = 231)		(n = 237)			(n = 70)		(n = 77)		(n = 78)		
Age, years	58.6	(11.2)	57.9	(10.7)	58.6	(11.8)	0.7306	57.2	(11.1)	61.4	(12.0)	60.3	(11.0)	0.0713
BMI	23.14	(4.09)	22.98	(4.27)	22.78	(4.02)	0.6382	22.85	(4.44)	23.42	(4.12)	23.83	(4.13)	0.3700
Duration of OAB symptom, month	59.6	(69.3)	73.0	(82.2)	60.5	(66.0)	0.0847	51.7	(51.4)	63.6	(61.7)	56.3	(46.4)	0.3963
Micturition number per 24 h	11.14	(2.28)	11.12	(2.19)	10.96	(2.13)	0.6287	11.00	(2.58)	11.33	(2.29)	12.08	(2.90)	0.0346
Urgency episodes per 24 h	3.68	(1.96)	3.86	(2.17)	3.72	(2.08)	0.6077	4.25	(1.90)	3.90	(1.94)	4.42	(2.57)	0.3253
Urgency urinary incontinence episodes per 24 h	1.86	(1.34)	1.77	(1.16)	1.73	(1.14)	0.4710	2.42	(1.79)	2.33	(1.67)	2.46	(1.67)	0.8898
Urinary incontinence per 24 h	1.86	(1.34)	1.77	(1.16)	1.73	(1.14)	0.4710	3.15	(2.01)	3.09	(2.12)	3.22	(1.98)	0.9222
Nocturia episodes per night	1.11	(0.89)	1.14	(0.95)	1.12	(0.86)	0.9349	1.29	(1.12)	1.16	(1.05)	1.40	(1.13)	0.4223
Voided volume per micturition, mL	155.74	(43.66)	154.35	(42.96)	158.09	(44.06)	0.6420	146.31	(45.88)	157.48	(49.10)	150.31	(46.46)	0.3461
KHQ domain score (n)	(n = 236)		(n = 231)		(n = 237)			(n = 70)		(n = 77)		(n = 78)		
D1: general health perception	28.92	(18.10)	28.90	(17.78)	26.79	(17.96)	0.3364	28.93	(19.33)	33.12	(16.94)	33.01	(16.85)	0.2695
D2: incontinence impact	48.16	(24.05)	47.76	(24.35)	48.38	(24.61)	0.9619	50.48	(24.57)	46.32	(21.73)	52.99	(25.45)	0.2190
D3: role limitations	40.68	(24.75)	40.84	(23.70)	41.49	(25.38)	0.9303	42.38	(20.98)	39.39	(24.17)	47.22	(26.24)	0.1248
D4: physical limitations	40.25	(27.49)	43.80	(26.69)	41.98	(26.95)	0.3683	41.90	(23.87)	39.39	(26.06)	50.00	(24.76)	0.0236
D5: social limitation	20.62	(21.81)	25.69	(23.26)	21.61	(22.84)	0.0385	22.22	(21.04)	17.46	(18.75)	25.36	(22.05)	0.0590
D6: personal relationships < n >	8.16	(17.14)	12.03	(19.69)	8.69	(17.28)	0.0794	9.18	(15.61)	7.34	(16.16)	13.69	(20.38)	0.1440
	< 192 >		< 187 >		< 188 >		-	< 49 >		< 59 >		< 56 >		-
D7: emotions	37.95	(22.91)	37.52	(24.91)	36.29	(24.85)	0.7418	40.00	(24.33)	35.50	(25.24)	42.17	(26.34)	0.2513
D8: sleep/energy	31.99	(25.16)	32.90	(26.55)	31.36	(24.52)	0.8060	31.43	(23.83)	31.17	(22.19)	36.97	(24.71)	0.2311
D9: severity measures < n >	38.76	(18.24)	40.06	(19.99)	38.87	(17.73)	0.7072	45.05	(16.85)	40.52	(17.99)	44.70	(17.36)	0.2078
	-		-		< 236 >		-	-	-	-	-	-	-	-

Effects on urinary parameters

**Figure 2:**
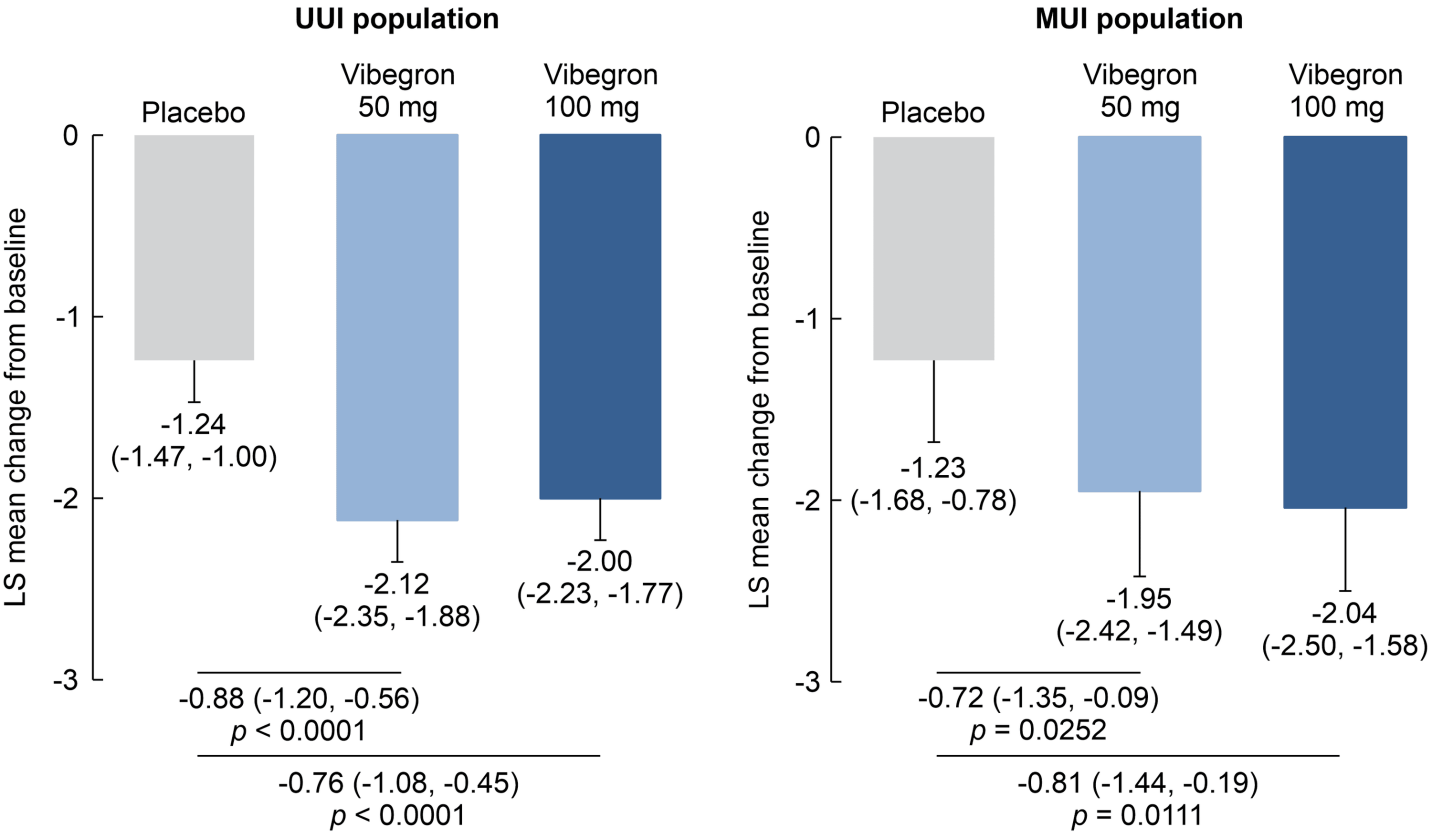
Change from baseline to week 12 in the mean micturition number per 24 h in the UUI and MUI populations. Data are presented as the mean (95% confidence interval). The differences were calculated using a constrained longitudinal data analysis model that included adjustment factors. UUI: Urgency urinary incontinence; MUI: Mixed urinary incontinence

The change from baseline to week 12 was calculated for each treatment group and compared to that of the placebo group. In both the UUI and MUI populations, the primary efficacy endpoint for OAB treatment, the mean micturition number per 24 hours, was significantly reduced in the vibegron 50 mg and 100 mg groups compared with that in the placebo group (Figure 2). Furthermore, in both the UUI and MUI populations, the mean changes in urgency episodes per 24 hours, incontinence episodes per 24 hours, and voided volume per micturition showed significant improvements in the vibegron 50 mg and 100 mg groups compared with that in the placebo group. However, only the vibegron 100 mg group demonstrated a significant improvement in nocturia episodes per night in both the UUI and MUI populations (Table 2).

**Table 2:** Change in the other endpoint parameters from baseline to week 12. UUI: Urgency urinary incontinence; MUI: Mixed urinary incontinence

	UUI population			MUI population		
	Vibegron 50 mg (n = 237)	Vibegron 100 mg (n = 231)	Placebo (n = 237)	Vibegron 50 mg (n = 70)	Vibegron 100 mg (n = 77)	Placebo (n = 78)
Urgency episodes per 24 h						
Least-square mean change	-2.34	-2.44	-1.84	-2.38	-2.83	-1.73
(95% Cl)	(-2.57, -2.11)	(-2.68, -2.21)	(-2.07, -1.61)	(-2.85, -1.90)	(-3.30, -2.36)	(-2.19, -1.27)
Difference vs. placebo	-0.50	-0.61	-	-0.64	-1.09	-
(95% Cl)	(-0.80, -0.20)	(-0.91, -0.30)	-	(-1.27, -0.01)	(-1.72, -0.47)	-
p-value	0.0013	0.0001	-	0.0461	0.0007	-
Incontinence episodes per 24 h						
Least-square mean change	-1.25	-1.27	-1.01	-2.04	-2.34	-1.36
(95% Cl)	(-1.38, -1.11)	(-1.41, -1.13)	(-1.14, -0.87)	(-2.24, -1.63)	(-2.74, -1.94)	(-1.75, -0.97)
Difference vs. placebo	-0.24	-0.26	-	-0.68	-0.98	-
(95% Cl)	(-0.42, -0.06)	(-0.44, -0.08)	-	(-1.21, -0.15)	(-1.51, -0.46)	-
p-value	0.0076	0.0039	-	0.0124	0.0003	-
Mean voided volume per micturition, mL						
Least-square mean change	36.94	30.94	7.10	29.17	31.22	12.13
(95% Cl)	(31.82, 42.06)	(25.76, 36.12)	(1.97, 12.24)	(19.49, 38.84)	(21.68, 40.76)	(2.81, 21.46)
Difference vs. placebo	29.84	23.84	-	17.03	19.09	-
(95% Cl)	(22.59, 37.09)	(16.55, 31.12)	-	(3.59, 30.48)	(5.73, 32.44)	-
p-value	<0.0001	<0.0001	-	0.0133	0.0053	-
Nocturia episodes per night (n)	(n = 198)	(n = 187)	(n = 201)	(n = 61)	(n = 63)	(n = 69)
Least-square mean change	-0.59	-0.61	-0.49	-0.63	-0.72	-0.45
(95% Cl)	(-0.68, -0.50)	(-0.71, -0.52)	(-0.58, -0.40)	(-0.78, -0.47)	(-0.88, -0.56)	(-0.60, -0.30)
Difference vs. placebo	-0.11	-0.13	-	-0.17	-0.26	-
(95% Cl)	(-0.22, 0.01)	(-0.25, -0.01)	-	(-0.37, 0.03)	(-0.47, -0.06)	-
p-value	0.0822	0.0399	-	0.0910	0.0102	-

The proportion of UI normalization at week 12 in the UUI population was 51.5% and 57.6% in the vibegron 50 mg and 100 mg groups, respectively, and the proportion in the vibegron 100 mg group was significantly higher than that in the placebo group (44.7%, p = 0.0054). In contrast, in the MUI population, the proportions in the vibegron 50 mg and 100 mg groups were 40.0% and 41.6%, respectively, both of which were significantly higher than those in the placebo group (20.5%, p = 0.0096 and p = 0.0046, respectively).

Changes in KHQ domain scores and PGI

**Figure 3:**
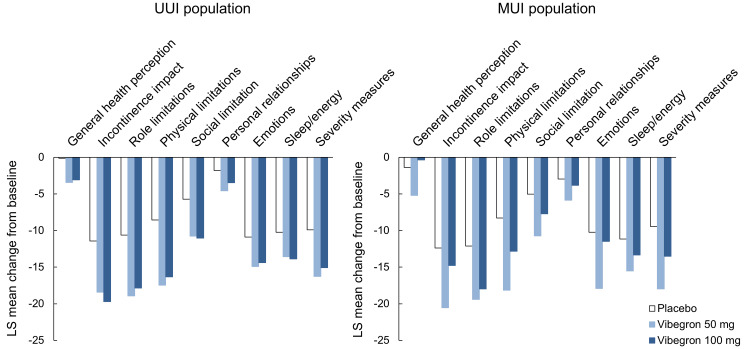
Change from baseline to week 12 in KHQ domain score in UUI and MUI populations. UUI: Urgency urinary incontinence; MUI: Mixed urinary incontinence; KHQ: King’s Health Questionnaire

In the QOL evaluation using the KHQ, vibegron improved all QOL items for both the UUI and MUI populations, with the exception of general health perception in the MUI population. The changes in these effects were comparable between the two populations (Figure 3). The PGI assessment revealed that most patients responded with “satisfied” in the UUI population (92.0% and 93.5% for the vibegron 50 mg and 100 mg groups, respectively) and in the MUI population (90.0% and 84.4%, respectively). The proportion of patients responding “very much satisfied” for the vibegron 50 mg and 100 mg groups was 60.8% and 64.5%, respectively, in the UUI population and 60.0% and 49.4%, respectively, in the MUI population. In both populations, the proportion of satisfied patients was significantly higher in the vibegron group than in the placebo group (Table 3).

**Table 3:** Assessment of treatment group-specific PGI for the UUI and MUI populations. PGI: Patient Global Impression; UUI: Urgency urinary incontinence; MUI: Mixed urinary incontinence

	UUI population			MUI population		
	Vibegron 50 mg (n = 237)	Vibegron 100 mg (n = 231)	Placebo (n = 237)	Vibegron 50 mg (n = 70)	Vibegron 100 mg (n = 77)	Placebo (n = 78)
Satisfied						
Proportion (%)	92.0	93.5	78.1	90.0	84.4	70.5
(95% Cl)	(87.8, 94.8)	(89.6, 96.0)	(72.4, 82.9)	(80.8, 95.1)	(74.7, 90.9)	(59.6, 79.5)
p-value	<0.0001	<0.0001	-	0.0032	0.0385	-
Very much satisfied						
Proportion (%)	60.8	64.5	42.2	60.0	49.4	25.6
(95% Cl)	(54.4, 66.8)	(58.1, 70.4)	(36.1, 48.6)	(48.3, 70.7)	(38.5, 60.3)	(17.3, 36.3)
p-value	0.0001	<0.0001	-	<0.0001	0.0023	-

Safety assessment

The overall incidence of TEAEs during the treatment period varied from 26.2% to 29.0% in the UUI population and from 26.9% to 41.6% in the MUI population. The incidence of drug-related TEAEs varied from 3.8% to 8.0% in the UUI population and from 2.9% to 7.7% in the MUI population. Drug-related TEAEs that occurred in ≥1% of the participants are shown in Table 4.

**Table 4:** Patients with treatment-emergent adverse event (FAS). AEs: Adverse events; TEAEs: Treatment-emergent adverse events; FAS: Full analysis set; UUI: Urgency urinary incontinence; MUI: Mixed urinary incontinence

	UUI population			MUI population		
	Vibegron 50 mg (n = 237)	Vibegron 100 mg (n = 231)	Placebo (n = 237)	Vibegron 50 mg (n = 70)	Vibegron 100 mg (n = 77)	Placebo (n = 78)
AEs, n (%)						
All	64 (27.0)	67 (29.0)	62 (26.2)	21 (30.0)	32 (41.6)	21 (26.9)
Drug-related TEAE	19 (8.0)	13 (5.6)	9 (3.8)	2 (2.9)	5 (6.5)	6 (7.7)
Most common drug-related TEAE (preferred term incidence ≥1% in any group)						
Anemia	0 (0.0)	0 (0.0)	0 (0.0)	0 (0.0)	1 (1.3)	1 (1.3)
Abdominal pain upper	0 (0.0)	0 (0.0)	0 (0.0)	0 (0.0)	1 (1.3)	1 (1.3)
Constipation	4 (1.7)	0 (0.0)	0 (0.0)	0 (0.0)	0 (0.0)	1 (1.3)
Dry mouth	2 (0.8)	1 (0.4)	1 (0.4)	1 (1.4)	0 (0.0)	1 (1.3)
Hepatic function abnormal	1 (0.4)	0 (0.0)	0 (0.0)	0 (0.0)	2 (2.6)	0 (0.0)
Cystitis	0 (0.0)	0 (0.0)	0 (0.0)	1 (1.4)	0 (0.0)	0 (0.0)
Blood creatine phosphokinase increased	0 (0.0)	1 (0.4)	1 (0.4)	0 (0.0)	1 (1.3)	0 (0.0)
Blood creatinine increased	0 (0.0)	0 (0.0)	0 (0.0)	0 (0.0)	1 (1.3)	0 (0.0)
Low-density lipoprotein increased	0 (0.0)	0 (0.0)	1 (0.4)	0 (0.0)	0 (0.0)	1 (1.3)
White blood cells urine positive	0 (0.0)	0 (0.0)	0 (0.0)	0 (0.0)	0 (0.0)	1 (1.3)
Residual urine volume increased	0 (0.0)	0 (0.0)	1 (0.4)	0 (0.0)	1 (1.3)	0 (0.0)
Drug-related serious TEAE	0 (0.0)	0 (0.0)	0 (0.0)	0 (0.0)	0 (0.0)	0 (0.0)

## Discussion

In this post hoc analysis, we compared the efficacy and safety of vibegron in female patients with UUI and MUI using bladder diary information obtained from a vibegron phase 3 study. It is important for doctors to confirm the types of incontinence: UUI, SUI, and MUI. However, in the phase 3 study, we did not use a questionnaire on SUI. Therefore, in this analysis, we classified the UUI and MUI populations according to bladder diary records. Notably, moderate agreement between the responses to self-reported questions on incontinence types and diary results has been reported [14].

In both the UUI and MUI populations, we found that vibegron (50 mg and 100 mg) significantly improved key parameters, including the number of micturition episodes per 24 hours, urgency episodes per 24 hours, and incontinence episodes per 24 hours, compared with placebo. Furthermore, vibegron improved all QOL items for patients in both populations (except for the "General Health Perception" item in the MUI population) and showed significantly higher treatment satisfaction compared to placebo in both populations. The effect of mirabegron, another β_3_-adrenoceptor agonist, on MUI in female patients was reported previously [15]. Although the classification methods for UUI and MUI were different, the effect of mirabegron on urgency episodes per 24 hours in the MUI population was not significant. However, the other results for vibegron in this analysis were almost the same as those reported for mirabegron.

It is unclear why β_3_-adrenoceptor agonists show similar effects in both UUI and MUI groups. For vibegron, this similarity may be attributable to one of the entry criteria for the vibegron phase 3 study, which stipulated that the total number of UUI episodes must exceed half of the total number of incontinence episodes. As an OAB treatment, vibegron may have improved OAB symptoms in the MUI group and thereby led to an overall improvement in storage symptoms. In reports evaluating mirabegron, the proposed mechanism suggested that mirabegron may improve bladder compliance during abdominal pressure increases and thus potentially prevent temporary excessive intravesical pressure increases [15]. Vibegron also improves bladder compliance in urodynamic studies of patients with neurogenic bladder [16,17]. Additionally, the basic pharmacological actions of β_3_-adrenoceptor agonists (e.g., inhibition of bladder smooth muscle contraction and suppression of acetylcholine release from parasympathetic cholinergic nerve terminals) may also contribute to the improvement of bladder compliance [18].

As UI significantly reduces patients’ QOL [2], we also assessed the changes in QOL using the KHQ and treatment satisfaction following vibegron administration. We found that KHQ score changes in the vibegron groups exceeded those of the placebo group in both the UUI and MUI populations for all items except for general health perception in the MUI population. The PGI assessment of vibegron treatment also demonstrated that the proportions of patients who were “very much satisfied” and “satisfied” were nearly equivalent between the UUI and MUI groups. These results indicate that vibegron improves QOL and treatment satisfaction equally in both the UUI and MUI populations and thus suggest that vibegron is an effective treatment option for MUI patients as well.

Regarding the safety profile of vibegron (50 mg and 100 mg), TEAEs that occurred in ≥2% of the patients included only hepatic functional abnormality with vibegron 100 mg in the MUI population, and there was no tendency for increased side effects in the MUI population compared with the UUI population.

The present study had some limitations. First, the number of patients classified with MUI was small. For items for which no statistical differences were observed, making judgments during trials with larger sample sizes is necessary. Second, because of the extremely small number of male patients included in the analysis, the analysis was conducted only on female patients. Consequently, the effects of vibegron on male MUI patients have not yet been evaluated. Third, the present analysis included a younger patient group than UI patients in the real world. The number of UI patients increases with age, with >30% of women aged 70 and above affected [19]. Therefore, further studies targeting older adults are warranted. Finally, further research is required to ascertain the efficacy and safety of vibegron in clinical settings with diverse patient populations, such as those with varying severities of UI and various comorbidities.

## Conclusions

This post hoc analysis of the vibegron phase 3 study confirmed the efficacy and safety of vibegron in patients with MUI. Vibegron therapy improved urinary parameters, patient QOL, and treatment satisfaction in participants with either UUI or MUI, without notable intergroup differences in drug-related TEAEs. Therefore, vibegron potentially constitutes an effective treatment option for patients with MUI.
